# Medical Opinion and Movement

**Published:** 1911-05-13

**Authors:** 


### Medical Opinion and Movement.

### The Tolerance of the Diabetic.

In a contribution to the *Medical Record* Dr. H. S. Carter lays down the principles which he adopts in the treatment of diabetes, especially with a view to increasing the carbohydrate tolerance, as it is called; that is, the amount of carbohydrate which can be consumed without producing glycosuria. In the first place it is necessary to find out, if possible, what the individual's tolerance is. This is done by putting the patient on von Noorden's standard diet; or at least restricting absolutely the carbohydrate intake to 100 grms. per day in the form of white bread. If after two days of this diet sugar is still being excreted, the amount of bread is reduced to 50 grms.; and in each instance the day's supply is divided equally between the three principal meals. If sugar still appears, the amount is further decreased by small decrements until the carbohydrate tolerance is reached. Then a diet is ordered containing a full protein ration, with large amounts of fatty foods, and with just half the tolerated quantity of carbohydrate. This low level is maintained for two or three months; then the toleration will usually be found to be considerably raised, but as it is not advisable actually to cause glycosuria again so as to estimate it, the better plan is to make a very moderate increase of the carbohydrates allowed. A table is constructed showing the equivalent carbohydrate value of a large number of articles of diet, thus allowing the patient a fairly varied menu. Thus 40 grms. of oatmeal is equivalent to 7.5 grms. of white bread; 25 grms. of peaches equals 3.5 grms. of white bread; 40 grms. of orange equals 7.5 grms. of white bread; and so on. Careful weighing of every carbohydrate foodstuff swallowed is essential.

### The Ætiology of Intussusception.

In a post-graduate lecture at Manchester reported for the *Clinical Journal* Mr. Stanmore Bishop lays great stress on the impropriety of cutting, dividing, and suturing the intestine in these cases; the infants are so young and their intestines so delicate that such proceedings are almost uniformly fatal. To avoid the necessity for intestinal interference the main thing is early diagnosis and early exploration in all suspicious or doubtful cases. The author attributes the occurrence of intussusception to developmental defects. In all his cases he has found the cæcum and ascending colon free to move, owing to their still possessing a mesentery similar to that of the small intestine instead of being firmly anchored to the right iliac fossa and posterior abdominal wall. The mere fact that the increase is at the expense of the ensheathing layer proves that this layer must be free to move. Following up this idea to its logical conclusion, he suggests and practises a plan for preventing the recurrence of intussusception, an event not by any means unknown. When once the colon and cæcum are anchored by the natural blending of their mesentery with the parietal peritoneum lining the posterior right abdominal wall, intussusception of this gut becomes, if not impossible, still tremendously hampered. It is not necessary to imitate the natural process to the full extent by attaching the whole peritoneal surfaces; it is sufficient to fix the cæcum to the right iliac fossa by two or three sutures, the rest being brought about by the weight of the superimposed small intestines.

### Saline Injections for Sciatica.

In the *Glasgow Medical Journal* Dr. Hay narrates his further experiences with saline injections into the sciatic nerve for obstinate cases of sciatica. In all he has now treated twelve patients, of whom eight have been cured, one was made worse, and three were lost sight of. As regards technique, he now injects at the sciatic foramen or at the gluteal fold, according as pressure causes greater pain at the one point or the other. To find the foramen, he draws imaginary straight lines from the posterior superior iliac spine to the top of the greater trochanter and to the middle of the ischial tuberosity; he then bisects the angle between them and takes a point two and a half inches along the bisecting line. At the gluteal fold the nerve lies midway between the trochanter and the tuberosity. To ascertain whether the needle has entered the nerve, press the plunger of the syringe gently so as to expel a few drops of the solution. If the needle is in the nerve the patient experiences a sensation as if something were trickling down within the leg. The usual amount for an injection is 10 c.c., repeated at intervals of three days. The author exhibited to the members of the local branch of the B.M.A. a patient formerly the subject of severe and recurrent sciatica who was cured by this saline-injection treatment, and has remained cured for eleven years.

### The Prevention of Flat Foot.

Some rules for the prevention of flat foot are formulated in the *London Hospital Gazette* by Dr. Paul Roth. Shoes should be worn, not slippers; they should have soles $\frac{1}{6}$ in. to $\frac{1}{4}$ in. in thickness, and broad heels 1 in. or less in height. The inner side of the shoe, where the big toe lies, must be kept almost straight; there must also be plenty of room for the other toes, but the big toe is the most important. When standing turn both toes slightly in and the heels slightly out; never stand on one foot to rest the other one. When walking let the feet point straight forwards, not outwards. When aching is first noticed, after standing some time, raise the heels just clear of the ground every now and then, sufficiently to bring all the muscles of the foot into action; in addition, stand with the feet very slightly turned over on the outer border. If marked flatness has already supervened, these directions alone will be insufficient. Two exercises must then be added, and are to be done every morning first thing, with the shoes off. One is to raise and lower the heels slowly a hundred times; that is, to rise on the toes, steadying the body with a hand on a mantelpiece or wall. The other is to sit on one chair with the foot projecting beyond the edge of another, the calf resting on the seat. Keeping the knee still, slowly circumduct the foot to its full extent, down, in, up, out; the toes to point slightly inwards all the time. This is to be done fifty times. These exercises to be continued for at least three months, and as long afterwards as there are any symptoms.

### Liquid Air for Skin Lesions.

In the Boston *Medical and Surgical Journal* Dr. Theodore C. Beebe reports on the use of liquid air for various affections of the skin. The range of usefulness includes all varieties of nævi, epitheliomata, lupus erythematosus, birthmarks, warts, calluses, and some forms of chronic inflammation, in fact, all those lesions for which *x*-rays, radium, and solid carbon dioxide have been used with more or less success. The author claims that liquid air is superior to solid carbon dioxide because it is much less painful and yields more satisfactory results. Birthmarks can be removed in almost every case in a way which causes hardly any pain and leaves practically no scar. For lesions on the face he considers this method of treatment is in every way the most satisfactory yet devised. He emphasises the importance, of course, of applying the treatment as early as possible in the cases of birthmarks, nævi, etc., while the tissues are soft, in order to secure the best results. Applications of liquid air can be made easily and safely to the mucous membrane of the mouth, and in one case several applications were made to the sclerotic coat of the eyeball without any harmful results.

### Adrenalin and the Circulation.

At the annual congress of the German Surgical Society, Dr. Holzbach gave an account of his experimental work to determine the effect of adrenalin on the heart and blood-vessels. He used frogs for his work, finding that the effects are the same in cold-blooded as in warm-blooded animals. Studying the effects upon a frog's heart isolated and artificially irrigated by a stream of blood, he finds that adrenalin acts as a violent poison, causing a considerable acceleration in the number of contractions, an incomplete diastole, a lowering of the blood-pressure, and a diminution in the work of the heart amounting to more than 75 per cent. At the end of a short time the heart stops in systole. The action is rapid and quickly passes off. If the heart of the animal is replaced by a pump, in order to study the circulatory system without the heart, adrenalin produces the same increase of blood pressure as in a sound animal. Furthermore, if in addition the brain and spinal cord are removed, the same effect is produced; therefore, the author argues, this increase of blood pressure is due to direct action on the peripheral vessels. Further experiments were made to determine the effect of adrenalin in morbid conditions, such as poisoning by arsenic. The isolated heart was subjected to the action of arsenic, causing a fall of blood pressure, diminution in the number of contractions, and finally arrest in diastole. In a short time adrenalin re-establishes the blood pressure, the number of contractions, and the function of the organ. Similarly in a rabbit, after almost completely annulling the blood pressure by means of arsenic, life was maintained for several hours by continuous injections of adrenalin. The author points out that these experiments demonstrate the dangers as well as the therapeutic value of adrenalin in conditions of low blood-pressure. Owing to the transitory action of the drug, the author advocates continuous intravenous injections in such cases.

### Ulcer of the Stomach.

At the same meeting Dr. Katzenstein returned to his favourite subject—the pathology of gastric ulcer. The stomach is preserved against auto-digestion under normal conditions by the antipepsin contained in its tissues. This substance is not only found in the walls of the stomach, but also in the blood. By implantations of intestine and of the spleen he has demonstrated that living tissues are digested by the stomach. The first condition for the formation of a gastric ulcer is a lesion in the mucous membrane caused by some disturbance in the circulation. Körte has shown that a lesion artificially produced in the gastric mucous membrane heals. The second condition is a failure of equilibrium between the normal proportions of pepsin and antipepsin. Dr. Katzenstein demonstrated by a number of preparations exhibited, that by causing a diminution of antipepsin in the blood and in the wall of the stomach he has been able to produce typical progressive and perforating ulcers. By further experiments he has found that whereas ordinary lesions of the gastric mucous membrane heal in about a week, ulcerations, in the neighbourhood of which the antipepsin is destroyed, take the course and form of a typical gastric ulcer.

### Extensive Skin Grafts.

In the *Bulletin of the Johns Hopkins Hospital* is printed the report of a case of very extensive burns, in which a great deal of skin grafting had to be done. The patient was a negro, burnt practically all over his back, and also slightly on one arm. The total area grafted, as measured after entire recovery, was two hundred and twelve square inches, nearly one sixth of the whole area of his body; the burnt area was, of course, a good deal larger still. At the first sitting a hundred and thirty Thiersch grafts, taken from the thighs, were applied; a hundred and ten of them "took," and altogether a very large surface was thus covered with skin. Then an experiment with amniotic membranes was tried, but failed completely. This is of interest in connection with the paragraph on this subject which we printed last week in this column (page 129). Next some isografts were tried—namely, pieces of skin taken from other patients—but none of these proved satisfactory, although applied under just the same conditions as the homografts. Ultimately, at three more sittings the remainder of the raw surface was covered by Thiersch grafts, and the final result was quite perfect. The grafts were applied direct to the granulations without curetting the latter; simple irrigation with salt solution was the only treatment of the granulations. The skin from which the grafts were cut was prepared by scrubbing with soap and water, rinsed off with alcohol and then with salt solution. Silver foil dressings were used, and left in place for ten days.

### Disseminated Sclerosis in a Young Boy.

It is generally agreed that disseminated sclerosis is very uncommon in children, so that particular interest attaches to the rapidly fatal case described by Dr. H. C. Mann in the *Guy's Hospital Gazette* as having occurred in a boy aged six. The patient, the youngest of a family of six, of whom all save himself were healthy, was the result of a natural labour and breast-fed. He had had measles and whooping-cough, and at the age of five went to school, where he was reported to be above the average in intellect for his years. In October 1909 he had a fit, apparently epileptic in type. Between then and June 1910 he seemed quite normal, but about the latter date he complained of feeling tired, and during the next two months became weak in the legs. By August, when he came under observation, he had developed a waddling gait, spoke slowly in monosyllables after a long latent period, but appeared interested in his surroundings. His facial expression was one of extreme boredom, suggesting mental deficiency at first. With the exception of increased knee-forks his reflexes were normal, and there was no muscle-wasting. Later he developed slight lateral nystagmus, and his legs became increasedly weak and his hands tremulous, though both grips were good. He was admitted to hospital in October. Examination of his eyes showed blurred discs suggesting neuritis, not atrophy; his legs were spastic and held in a position of talipes equino-varus. Knee-jerks were exaggerated and ankle-clonus was present, more marked on the right side. Plantar reflexes were definitely flexor. His gait was spastic and ataxic, with scissor-legged progression, the feet being dragged. Romberg's sign was absent. Electrical reactions of the lower extremities were normal. Six weeks later he was unable to stand or walk without support, intention tremor was well-marked in the arms, movements of head and neck were irregular, and mental deterioration was rapid. Early in December he became stuporose, had irregular epileptiform twitchings of limbs, marked anæsthesia of the lower limbs and periods of cyanosis. He died on December 25, after losing much in weight. Post-mortem examination showed sclerosis in the lateral and posterior columns of the cord.

### Appendicectomy under Cocaine.

Charles R. Hancock, of New York, reports in the *Medical Record* a series of seven operations for appendicitis carried out under cocaine analgesia. The following is the technique he employs: A solution of 1 in 200 cocaine is procured and injected subcutaneously and endermically. Then an incision three inches long is made one inch below the umbilicus and half an inch from the middle line and parallel to it. This incision is carried down to the sheath of the rectus. The injection is next made again into the sheath and into the rectus muscle. The peritoneum being insensitive, is not cocainised. The author states that the removal of adhesions is painless, and that the patient does not object to pulling on the viscera.

### Intravenous Injections of Cocaine.

The *Boston Medical and Surgical Journal* contains an article by P. W. Harrison on the effects of intravenous injection of cocaine (Biers' method). The amount used was 5 grains of the drug in a 2-per-cent. solution, and it took thirty minutes to complete the introduction. As the patient complained of dizziness and palpitation, no further amount of cocaine was given. The patient's condition was then carefully tested, and it was found that motor-power was quite unaffected, though the patient was unable to keep his mind for long on any one subject. Everywhere marked analgesia was found, and on making an incision through the skin well down into the fat on the anterior surface of the leg, the patient merely complained of trifling pain. On cutting two or three small nerves in the fat the patient twitched slightly, but it was obvious that operative measures could have been carried out with only trifling discomfort. Two hours later another incision was made on the opposite side, when it was found that most of the sensation of pain had returned. The author thinks that the relatively enormous dose necessary for even an imperfect result makes the method quite impracticable for surgical purposes. The experiment is of value, however, as it was carried out by the author on himself.

### Asphyxia after Lumbar Puncture.

Dr. Edward M. Colie, of New York, reports in the *Medical Record* a case of sudden death following upon spinal puncture for purposes of diagnosis. The patient, an infant of sixteen months, was in a state of coma when the operation was carried out. Soon after it became asphyxiated, and after periods of partial recovery finally died a few hours later. At the autopsy it was found to be suffering from purulent meningitis with a left-sided frontal lobe abscess. Death was the result of pressure on the respiratory centre caused by removal of fluid from the spinal canal, which was not in communication with the ventricles of the brain. The periods of recovery were due to the accumulated carbon dioxide in the blood re-establishing for a time the activity of the respiratory centre.

